# Effect of the decision to perform hysteroscopy on asymptomatic patients before undergoing assisted reproduction technologies—a systematic review and meta-analysis

**DOI:** 10.1016/j.xagr.2023.100178

**Published:** 2023-02-08

**Authors:** Greg J. Marchand, Ahmed Taher Masoud, Hollie Ulibarri, Julia Parise, Amanda Arroyo, Catherine Coriell, Sydnee Goetz, Carmen Moir, Atley Moberly

**Affiliations:** 1 Faculty of Medicine, Marchand Institute for Minimally Invasive Surgery, Mesa, AZ (Drs Marchand and Masoud, Mses Ulibarri, Parise, and Arroyo, Ms Coriell, and Mses Goetz, Moir, and Moberly); 2 Faculty of Medicine, Fayoum University, Fayoum, Egypt (Dr Masoud).

**Keywords:** assisted reproductive technology, hysteroscopy, infertility, in vitro fertilization

## Abstract

**OBJECTIVE:**

Routine hysteroscopic evaluation before assisted reproductive technology treatment is a novel approach with the potential to reduce assisted reproductive technology failure even in the absence of evidence of uterine pathology. Following the publication of several relatively high-quality trials on this topic, we sought to determine if this practice is beneficial.

**DATA SOURCES:**

We searched Web of Science, MEDLINE, PubMed, Scopus, Cochrane Library, and ClinicalTrials.gov from each database's inception until May 31, 2022 with our search strategy, attempting to locate all randomized controlled trials assessing the use of hysteroscopy in otherwise asymptomatic women undergoing assisted reproductive technology.

**STUDY ELIGIBILITY CRITERIA:**

We included only randomized controlled trials that included at least one of our selected outcomes, and we excluded any studies with suspicion of pathology before the time of hysteroscopy, other than knowledge of the patient's infertility. We included all the aforementioned studies regardless of procedures or modifications performed as a result of hysteroscopic findings. Our initial search yielded 1802 results, which were reduced to 1421 after removal of duplicates. Ultimately, 11 studies were found to meet our criteria and were included in our quantitative synthesis.

**METHODS:**

We used ReviewManager software, version 5.4.1 to analyze the data, which we imported after manually gathering from the 11 studies. Continuous and dichotomous outcomes were imported as standard deviations. Pooled analysis was described as a mean difference, relative to 95 % confidence interval in cases of continuous data. Dichotomous outcomes were analyzed using risk ratios and 95% confidence intervals. In homogeneous outcomes, we used a fixed-effects model, and in heterogeneous outcomes we used a random-effects model.

**RESULTS:**

Our results showed that hysteroscopy was associated with significant improvement in the clinical pregnancy rate (risk ratio, 1.27 [1.11–1.45]; *P*<.001). We found no differences between the hysteroscopy group and the control group in live birth rate (risk ratio, 1.26 [0.99–1.59]; *P*=.06), miscarriage rate (risk ratio, 0.99 [0.81–1.19]; *P*=.88), fertilization rate (risk ratio, 1.01 [0.93–1.09]; *P*=.88), incidence of multiple gestations (risk ratio, 1.29 [0.98–1.71]; *P*=.07), number of embryos transferred (mean difference, 0.04 [−0.18 to 0.26]; *P*=.73), chemical pregnancy rate (risk ratio, 1.01 [0.86–1.17]; *P*=.93), and number of oocytes retrieved (mean difference, 0.44 [−0.11 to 0.98]; *P*=.11).

**CONCLUSION:**

We observed an improvement in the clinical pregnancy rate, but no significant improvement in the live birth rate with routine hysteroscopy before assisted reproductive technology treatment. We believe this does not represent sufficient evidence to recommend routine hysteroscopy for otherwise asymptomatic patients before assisted reproductive technology treatment at this time.

**AJOG Global Reports at a Glance**

**Why was this study conducted?**

A: Despite no widely accepted guideline, our researchers noticed the publication of many studies where reproductive endocrinologists would routinely perform hysteroscopy prior to assisted reproduction technologies, such as in vitro fertilization, on asymptomatic patients with no identified pathology. As a result we sought out to find if evidence existed of a benefit to this practice.

**Key findings**

A: The decision to perform hysteroscopy prior to assisted reproduction technologies seems to be associated with a higher clinical pregnancy rate, but no difference was seen in the rates of live birth, the miscarriages rate, the incidence of multiple gestations or the chemical pregnancy rate.

**What does this add to what is known?**

This study does not definitively show benefit to the routine practice of hysteroscopy prior to assisted reproduction technologies, but adds to the body of evidence suggesting that there may be some improvement with this practice, as evidenced by the increased rate in clinical pregnancies.

## Introduction

Infertility, defined as the inability to achieve pregnancy after 1 year of unprotected, timed intercourse, can result in severe psychological, mental, and even medical disease for patients.^1^^,^^2^ It affects approximately 37% of couples worldwide, and originates from the male factor in 57% of cases, from the female factor in 35%, and from both in the remaining 8%.^3^ The most common causes of female-factor infertility include tubal blockage, ovulatory disorders, endometriosis, tubal abnormalities, uterine abnormalities, and hormonal disorders.^4^ A basic workup for infertility may include assessment of semen, ovarian reserve and function, the uterine cavity, the patency of the fallopian tubes, and endocrinology.^5^ Regarding the assessment of the uterine cavity, several options exist, including ultrasonography, hysterosalpingography, and hysteroscopy.^6^ Some clinicians may also choose to forgo the assessment in asymptomatic women.^6^ Although invasive, hysteroscopy provides the surgeon the possibility of immediate surgical repair of discovered pathology, which may include endometrial polyps, leiomyomas, septums, or other intrauterine pathology.^7^, ^8^, ^9^ In the recent months, we noticed several relatively high-quality randomized controlled trials (RCTs) published on the topic of performing hysteroscopy before assisted reproductive technology (ART) treatment in otherwise asymptomatic women. Thus, in this meta-analysis we sought to estimate the efficacy of performing hysteroscopy before ART application in improving the outcomes of the different ART techniques in infertile patients, and any resulting treatments or treatment plan modifications made as a result of the findings of that hysteroscopy.

## Methods

### Search strategy

This study was conducted in accordance with PRISMA (Preferred Reporting Items for Systematic Reviews and Meta-Analyses) guidelines.^10^ We conducted our search in electronic databases, including Web of Science, MEDLINE, PubMed, Scopus, Cochrane Library, and ClinicalTrials.gov, from the inception of each database until May 31, 2022 using the following strategy: (Infertility OR Sterility OR Subfertility OR Sub-Fertility) AND (Hysteroscop* OR “Uterine Endoscopy” OR ureteroscopy) AND (“live birth” OR “pregnancy rate” OR miscarriage).

### Study selection

Two authors performed title and abstract screening followed by full-text screening. This process was performed according to the following eligibility criteria:

• Population: infertile women undergoing any ART technique.
• Intervention: hysteroscopy in otherwise asymptomatic women.
• Comparator: control group.
• Outcomes: clinical pregnancy rate (defined as ultrasound and serologic confirmation of intrauterine pregnancy), live birth rate per cycle, miscarriage rate, fertilization rate, multiple pregnancies, number of transferred embryos, chemical pregnancy rate, and number of oocytes retrieved.
• Study design: we included RCTs only and excluded all other study designs, meta-analyses, and reviews.

### Quality assessment

We evaluated the risk of bias of the included RCTs according to the Cochrane Handbook for Systematic Reviews of Interventions.^11^ We assessed 7 domains in each study: (1) random sequence generation; (2) selective reporting; (3) blinding of participants and personnel; (4) blinding of outcome assessment; (5) incomplete outcome data; (6) allocation concealment; and (7) other biases.

### Data extraction

Data were retrieved manually from the included studies and placed into spreadsheets. We extracted baseline data such as the demographic data of patients, the number of patients with primary infertility, the number of patients with secondary infertility, the duration of infertility, and the causes of infertility. Then, we extracted the following outcomes: clinical pregnancy rate, live birth rate per cycle, miscarriage rate, fertilization rate, multiple pregnancies, number of transferred embryos, chemical pregnancy rate, and number of oocytes retrieved. We also extracted additional data that were required to complete our quality assessment of the included studies.

### Statistical analysis

We used ReviewManager (RevMan) software, version 5.4.1 (Cochrane, London, United Kingdom) to analyze the data. Continuous and dichotomous outcomes were imported from the spreadsheet into the RevMan software as mean±standard deviation and percentage and total, respectively. Pooled analysis was described as mean difference (MD), relative to 95% confidence interval (CI) in cases of continuous data, and dichotomous data were analyzed using risk ratio (RR) and 95% CI. In homogeneous outcomes, we used a fixed-effects model, whereas heterogeneous outcomes were analyzed under the random-effects model. We measured heterogeneity among studies using I-squared (Higgins *I*^2^). Outcomes with *I*² >50% or *P*<.1 in the pooled analysis were considered heterogeneous.^12^^,^^13^

## Results

### Summary of the included studies

**Figure 1:**
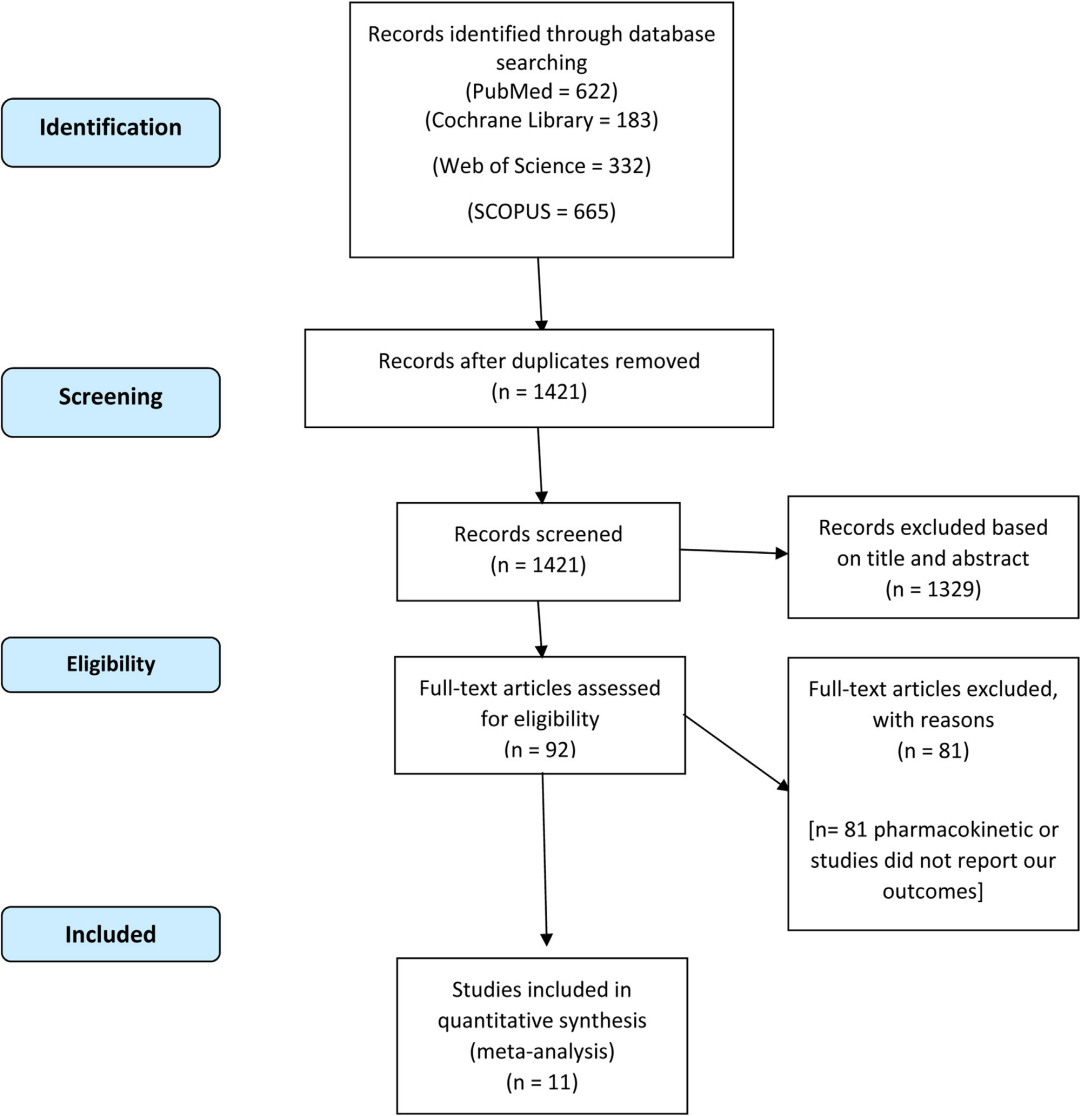
PRISMA flow diagram of our literature search PRISMA, Preferred Reporting Items for Systematic Reviews and Meta-Analyses.

The PRISMA flow diagram of our search is shown in Figure 1. We analyzed 3938 infertile women from 11 included RCTs^14^, ^15^, ^16^, ^17^, ^18^, ^19^, ^20^, ^21^, ^22^, ^23^, ^24^; 1821 patients underwent hysteroscopy, whereas 2117 patients were allocated to the control group. The 2 groups were found to be similar in sample size, age, and body mass index. The demographic data of patients, the number of patients with primary infertility, the number of patients with secondary infertility, the duration of infertility, and the causes of infertility are described in Table 1, Table 2, Table 3.

Marchand. Effect of the decision to perform hysteroscopy on asymptomatic patients. Am J Obstet Gynecol Glob Rep 2023.

**Table 1:** Demographic data of included patients

Study ID	Sample size		Age, y, mean (SD)		BMI, mean (SD)	
	Hysteroscopy	Control	Hysteroscopy	Control	Hysteroscopy	Control
Alleyassin et al,^14^ 2017	110	110	29.5 (3.8)	29.1 (4.3)	25.47 (3.71)	25.6 (3.7)
Ben Abid et al,^16^ 2021	84	87	32.3 (4.4)	32.3 (5.1)	NR	NR
Demirol and Gurgan,^15^ 2004	211	211	35.8 (0.4)	34.3 (0.8)	NR	NR
El-nashar and Nasr,^17^ 2011	62	62	NR	NR	NR	NR
Elsetohy et al,^18^ 2015	102	101	31.1 (5.8)	29.9 (4.8)	29.1 (5.0)	29.5 (5.8)
El-Toukhy et al,^19^ 2016	350	352	33 (0.685)	33 (0.686)	22.875 (1)	22.85 (0.8)
Ghasemi et al,^20^ 2022	123	125	29.9 (3.8)	30.5 (4.2)	24.19 (2.8)	23.9 (3.5)
Kilic et al,^21^ 2013	100	398	31.9 (3.4)	31.4 (3.2)	NR	NR
Moramezi et al,^22^ 2012	55	33	28.8 (3)	29.8 (3)	23.99 (0.59)	24 (0.69)
Rama Raju et al,^23^ 2006	255	265	28.2 (0.76)	26.7 (0.5)	22.9 (0.56)	26.7 (0.1)
Smit et al,^24^ 2016	369	373	33 (4.4)	33 (4.5)	25 (4.2)	24 (4.3)

*BMI*, body mass index; *NR*, not reported; *SD*, standard deviation.

**Table 2:** Patients with primary and secondary infertility, and the duration of infertility

Study ID	Primary infertility, n (%)		Secondary infertility, n (%)		Duration of infertility, y, mean (SD)	
	Hysteroscopy	Control	Hysteroscopy	Control	Hysteroscopy	Control
Alleyassin et al,^14^ 2017	NR	NR	NR	NR	4.74 (3.44)	4.59 (3.25)
Ben Abid et al,^16^ 2021	77 (91.7)	76 (87.4)	7 (8.3)	11 (12.6)	4.28 (2.99)	4.75 (3.43)
Demirol and Gurgan,^15^ 2004	NR	NR	NR	NR	6.1 (0.4)	6.2 (0.3)
El-nashar and Nasr,^17^ 2011	NR	NR	NR	NR	NR	NR
Elsetohy et al,^18^ 2015	58 (59.8)	65 (67.7)	39 (40.2)	31 (32.3)	5.9 (3.7)	5.7 (3.4)
El-Toukhy et al,^19^ 2016	NR	NR	NR	NR	4 (0.34)	3.75 (0.5)
Ghasemi et al,^20^ 2022	NR	NR	NR	NR	NR	NR
Kilic et al,^21^ 2013	NR	NR	NR	NR	7	6
Moramezi et al,^22^ 2012	NR	NR	NR	NR	3.7 (0.49)	4.4 (0.57)
Rama Raju et al,^23^ 2006	NR	NR	NR	NR	7.03 (0.62)	7.01 (0.10)
Smit et al,^24^ 2016	NR	NR	NR	NR	2.6 (1.9)	2.1 (2.7)

*NR*, not reported; *SD*, standard deviation.

**Table 3:** Causes of infertility in included patients

	Cause of infertility, %							
	Male factor		Ovulatory disorder		Tubal/peritoneal factor		Unexplained	
Study ID	Hysteroscopy	Control	Hysteroscopy	Control	Hysteroscopy	Control	Hysteroscopy	Control
Alleyassin et al,^14^ 2017	37 (33.6)	32 (29.1)	33 (30)	35 (32.1)	22 (20.1)	26 (23.5)	18 (16.3)	17 (15.3)
Ben Abid et al,^16^ 2021	70 (83.33)	75 (86.2)	NR	NR	7 (8.33)	2 (2.29)	2 (2.38)	4 (4.59)
Demirol and Gurgan,^15^ 2004	62 (29)	50 (24)	66 (31)	74 (35)	NR	NR	40	87 (41)
El-nashar and Nasr,^17^ 2011	NR	NR	NR	NR	NR	NR	NR	NR
Elsetohy et al,^18^ 2015	48 (49.5)	51 (53.1)	15 (15.5)	17 (17.7)	27 (27.8)	25 (26)	21 (21.6)	19 (19.8)
El-Toukhy et al,^19^ 2016	157 (45%)	159 (45%)	21 (6%)	26 (7%)	61 (17%)	53 (15%)	52 (15%)	62 (18%)
Ghasemi et al,^20^ 2022	NR	NR	NR	NR	NR	NR	NR	NR
Kilic et al,^21^ 2013	43 (43%)	173 (43%)	6 (6%)	31 (8%)	7 (7%)	37 (9%)	44 (44%)	157 (40%)
Moramezi et al,^22^ 2012	45 (80%)	36 (69.1%)	NR	NR	NR	NR	8 (14.8%)	15 (27.3%)
Rama Raju et al,^23^ 2006	NR	NR	NR	NR	NR	NR	NR	NR
Smit- et al,^24^ 2016	209 (57%)	193 (52%)	NR	NR	32 (9%)	40 (11%)	112 (30%)	104 (28%)

*NR*, not reported.

### Results of the quality assessment

**Figure 2:**
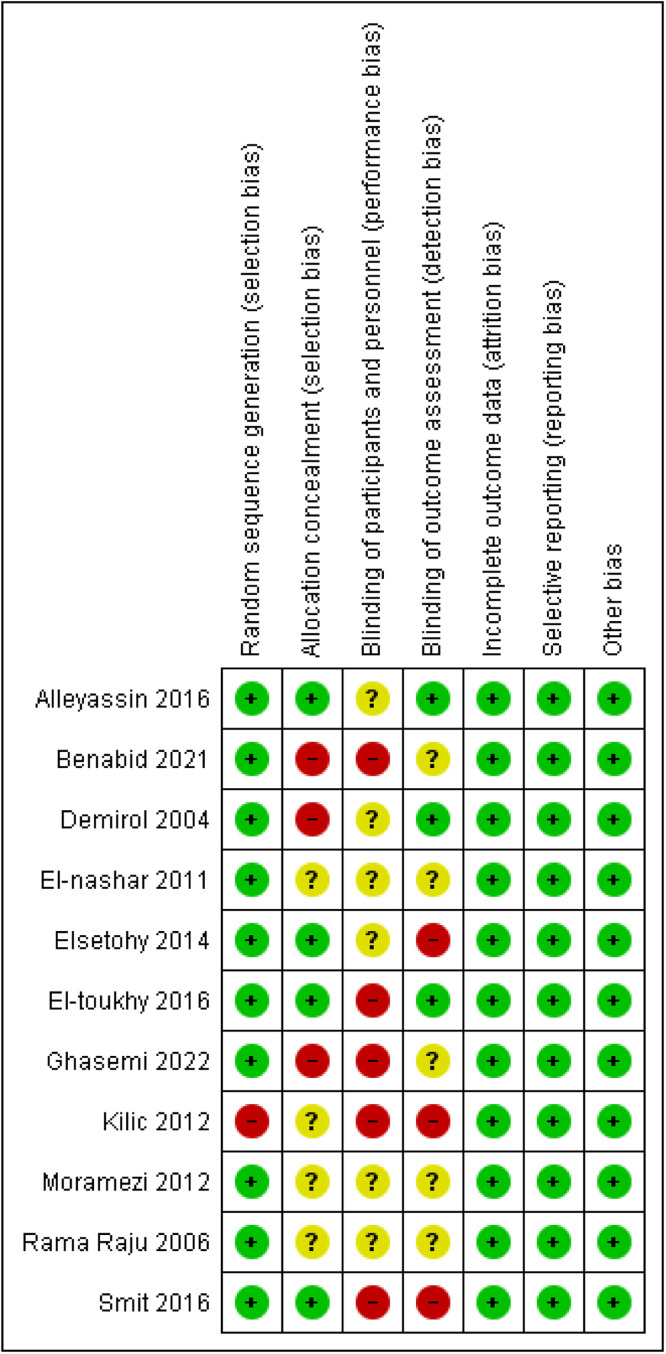
Full results of our quality assessments of the included studies

The quality assessment of the included trials yielded an overall moderate risk of bias. Concerning the randomization domain, all studies reported proper randomization, so they were categorized as having low risk of bias except Kilic et al,^21^ which was categorized as at high risk of bias. Regarding attrition and reporting bias, all studies were categorized as having low risk of bias. The full results of our assessment of quality are illustrated in Figure 2.

Marchand. Effect of the decision to perform hysteroscopy on asymptomatic patients. Am J Obstet Gynecol Glob Rep 2023.

### Analysis of outcomes

#### Clinical pregnancy rate

**Figure 3:**
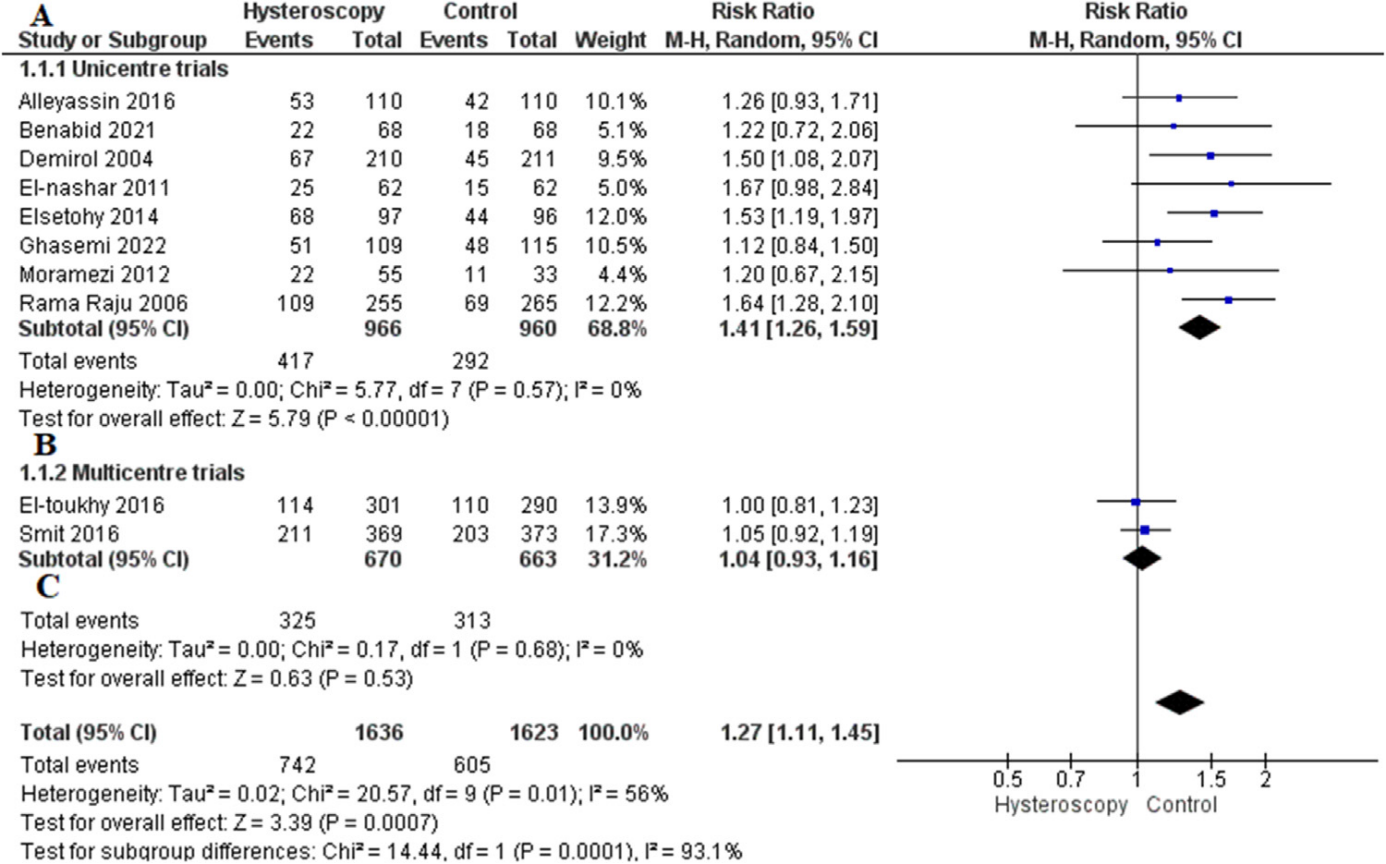
Forest plot of the outcome of rate of clinical pregnancy *CI*, confidence interval; *M-H*, Mantel–Haenszel.

Most studies reported this outcome^14^, ^15^, ^16^, ^17^, ^18^, ^19^, ^20^^,^^22^, ^23^, ^24^; 8 RCTs were conducted in 1 center and were allocated to the first subgroup (unicenter subgroup). The overall RR showed that hysteroscopy significantly increased the clinical pregnancy rate compared with the control group (RR, 1.41 [1.26–1.59]; *P*<.001). Data were homogeneous (*P*=.57; *I*²=0%) (Figure 3, A).

Marchand. Effect of the decision to perform hysteroscopy on asymptomatic patients. Am J Obstet Gynecol Glob Rep 2023.

Regarding the multicenter subgroup, data from 2 trials were analyzed. The combined RR did not show any difference between the hysteroscopy and the control group (RR, 1.04 [0.93–1.16]; *P*=.53). Pooled analysis was homogeneous (*P*=.68; *I*²=0%) (Figure 3, B).

The combined analysis of all trials from both subgroups favored the hysteroscopy group over the control group (RR, 1.27 [1.11–1.45]; *P*<.001) (Figure 3, C).

#### Live birth rate per cycle

**Figure 4:**
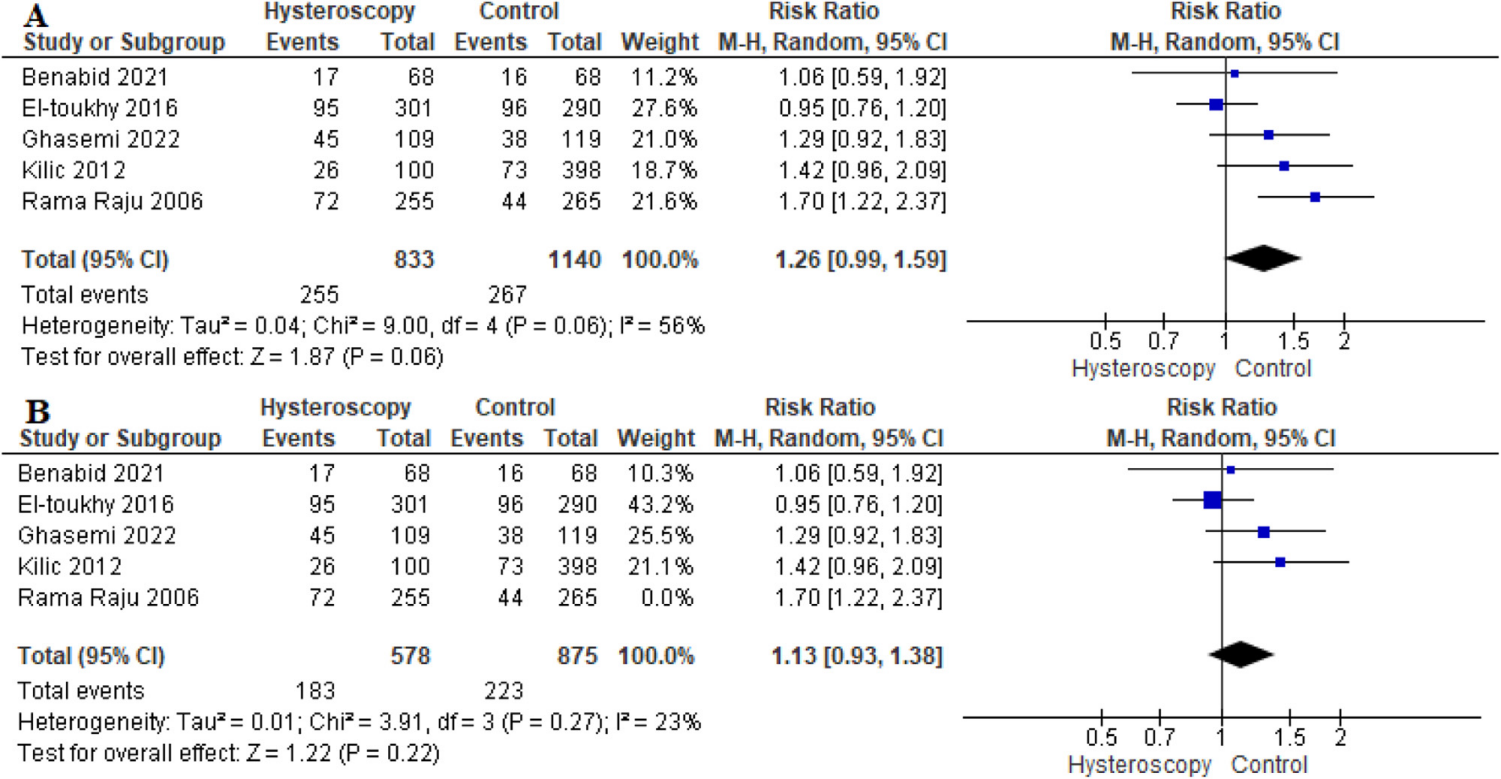
Forest plot of the live birth rate per cycle *CI*, confidence interval; *M-H*, Mantel–Haenszel.

Five studies reported the live birth rate as an outcome.^16^^,^^19^, ^20^, ^21^^,^^23^ The overall RR showed no significant difference between the 2 groups (RR, 1.26 [0.99–1.59]; *P*=.06). Data were heterogeneous (*P*=.06; *I*²=56%) (Figure 4, A). We were able to solve the heterogeneity by excluding Raju et al^23^ (*P*=0.27; *I*²=23%). The combined analysis after solving the heterogeneity also showed similar live birth rates between the 2 groups (RR, 1.13 [0.93–1.38]; *P*=.22) (Figure 4, B).

Marchand. Effect of the decision to perform hysteroscopy on asymptomatic patients. Am J Obstet Gynecol Glob Rep 2023.

#### Miscarriage rate

**Figure 5:**
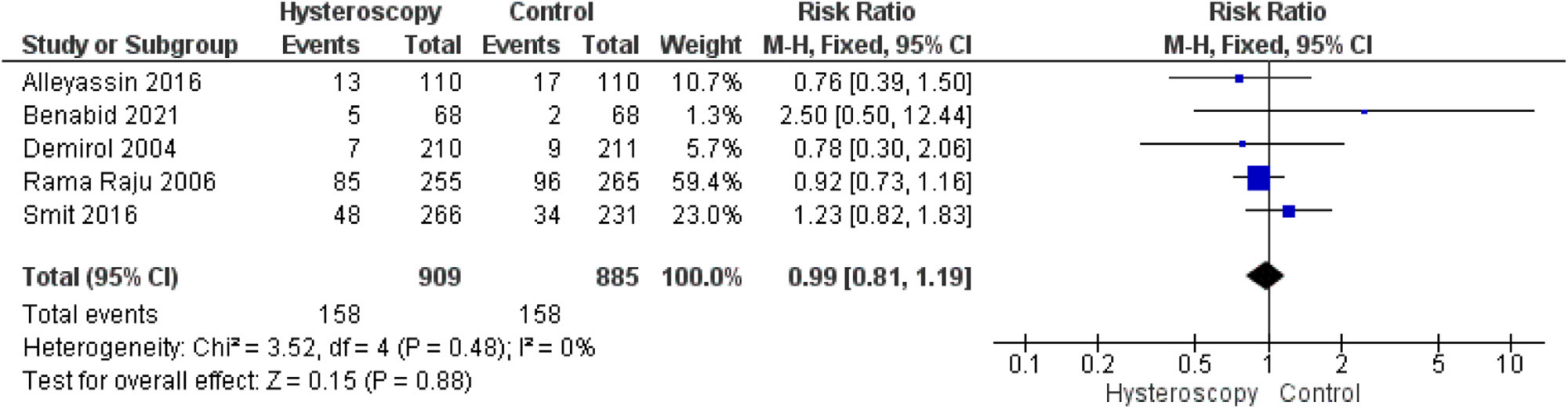
Forest plot of the miscarriage rate *CI*, confidence interval; *M-H*, Mantel–Haenszel.

Data on the miscarriage rate were retrieved from 5 studies.^14^, ^15^, ^16^^,^^23^^,^^24^ We found no significant difference between the hysteroscopy and the control group (RR, 0.99 [0.81–1.19]; *P*=.88). Pooled analysis was homogeneous (*P*=.48; *I*²=0%) (Figure 5).

Marchand. Effect of the decision to perform hysteroscopy on asymptomatic patients. Am J Obstet Gynecol Glob Rep 2023.

#### Fertilization rate

**Figure 6:**
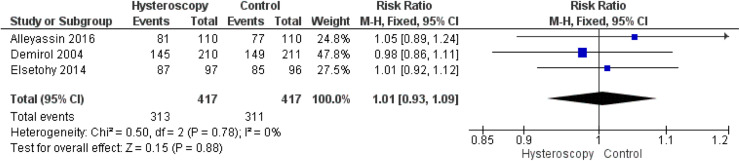
Forest plot of the fertilization rate *CI*, confidence interval; *M-H*, Mantel–Haenszel.

Three studies reported the fertilization rate.^14^^,^^15^^,^^18^ The overall RR showed a similar fertilization rate in both groups (RR, 1.01 [0.93–1.09]; *P*=.88). The overall analysis was homogeneous (*P*=.78; *I*²=0%) (Figure 6).

Marchand. Effect of the decision to perform hysteroscopy on asymptomatic patients. Am J Obstet Gynecol Glob Rep 2023.

#### Multiple pregnancy

**Figure 7:**
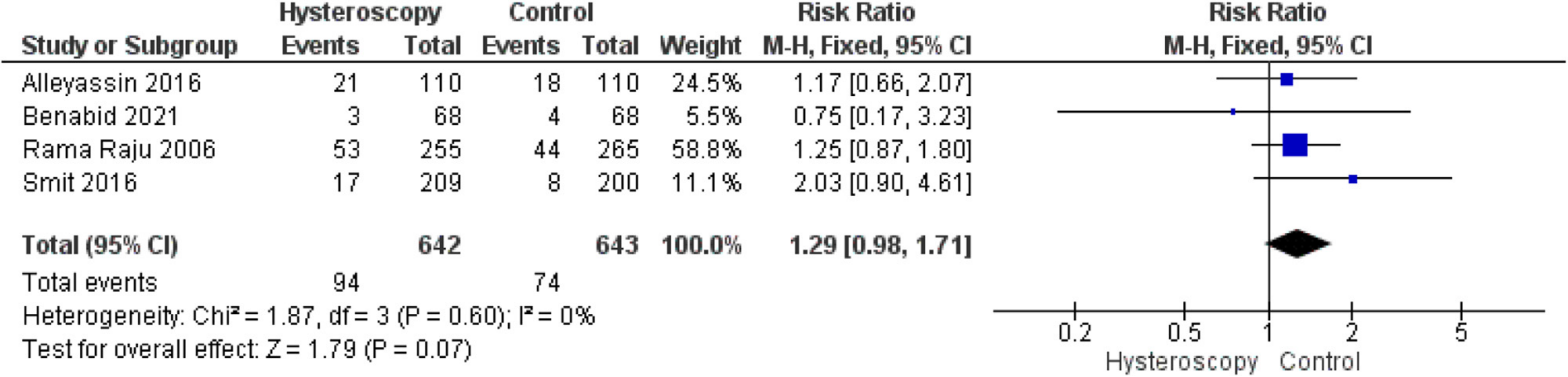
Forest plot of the rate of multiple pregnancy *CI*, confidence interval; *M-H*, Mantel–Haenszel.

Four studies^14^^,^^16^^,^^23^^,^^24^ reported the outcome of multiple pregnancy. The combined analysis showed no difference between the hysteroscopy and the control group (RR, 1.29 [0.98–1.71]; *P*=.07). Data were homogeneous (*P*=.60; *I*²=0%) (Figure 7).

Marchand. Effect of the decision to perform hysteroscopy on asymptomatic patients. Am J Obstet Gynecol Glob Rep 2023.

#### Number of transferred embryos

**Figure 8:**
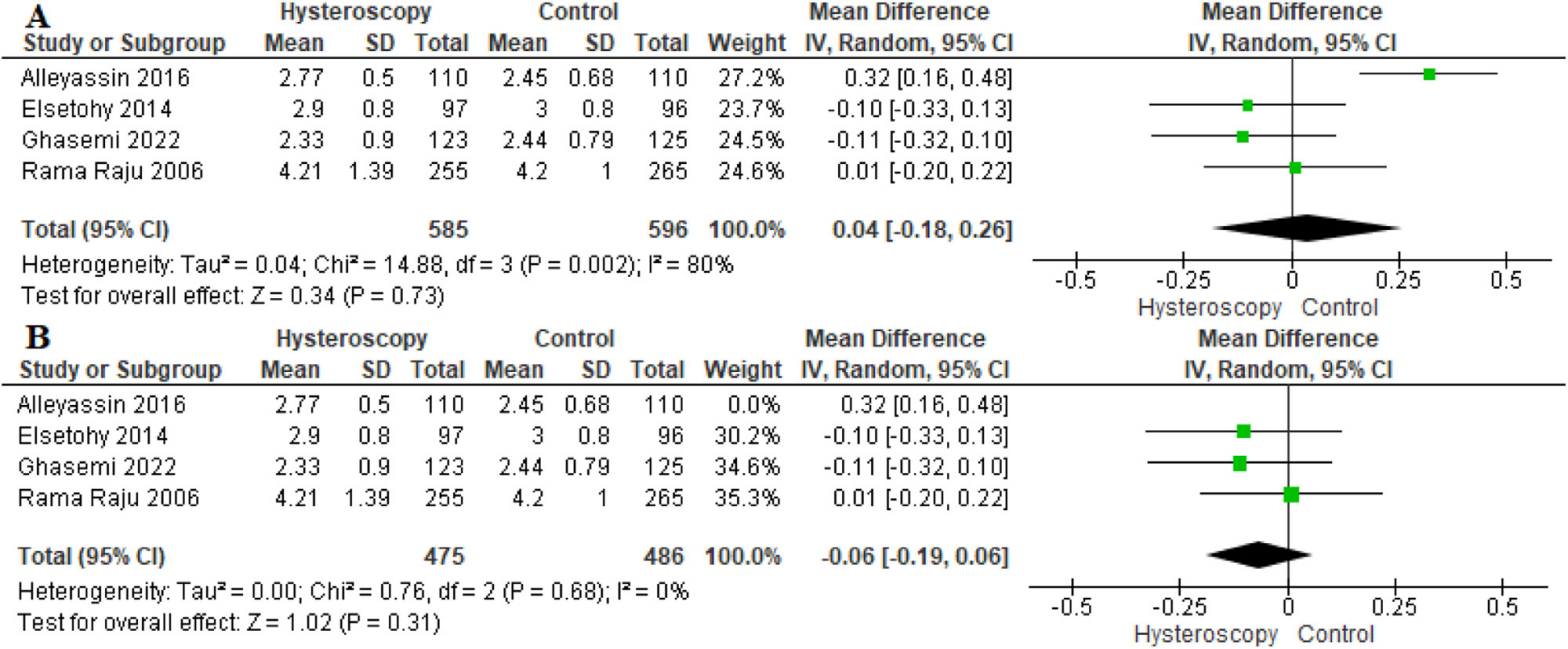
Forest plot of the number of embryos transferred *CI*, confidence interval; *IV*, inverse variance; *SD*, standard deviation.

Four studies reported data on the number of transferred embryos. We found no variation between the 2 groups (MD, 0.04 [−0.18 to 0.26]; *P*=.73). The overall MD was initially heterogeneous (*P*=.002; *I*²=80%) (Figure 8, A). To solve the heterogeneity among studies, we excluded Alleyassin et al^14^ (*P*=.68; *I*²=0%). The combined MD after solving the heterogeneity also showed a similar number of transferred embryos in both groups (MD, −0.06 [−0.19 to 0.06]; *P*=.31) (Figure 8, B).

Marchand. Effect of the decision to perform hysteroscopy on asymptomatic patients. Am J Obstet Gynecol Glob Rep 2023.

#### Chemical pregnancy rate

**Figure 9:**
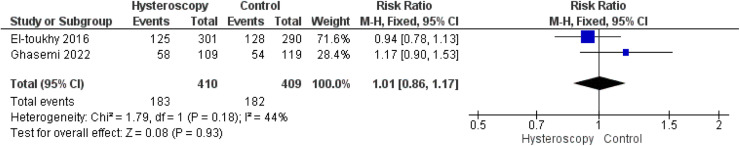
Forest plot of the chemical pregnancy rate *CI*, confidence interval; *M-H*, Mantel–Haenszel.

This outcome was reported by 2 studies.^19^^,^^20^ Pooled analysis showed no significant variation between the 2 groups (RR, 1.01 [0.86–1.17]; *P*=.93). The overall RR was homogeneous (*P*=.18; *I*²=44%) (Figure 9).

Marchand. Effect of the decision to perform hysteroscopy on asymptomatic patients. Am J Obstet Gynecol Glob Rep 2023.

#### Number of oocytes retrieved

**Figure 10:**
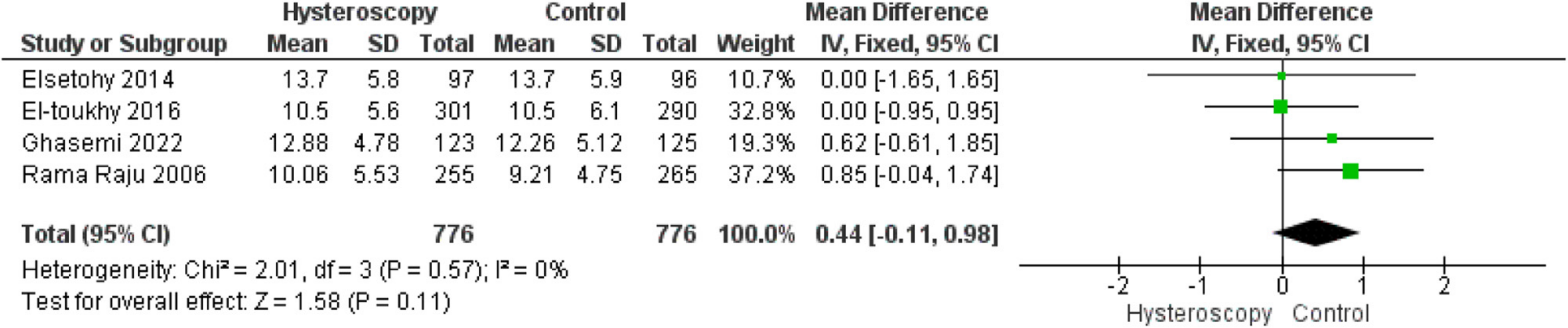
Forest plot of the number of oocytes retrieved *CI*, confidence interval; *IV*, inverse variance; *SD*, standard deviation.

The number of oocytes retrieved from patients was similar in both groups (MD, 0.44 [−0.11 to 0.98]; *P*=.11). Pooled data were homogeneous (*P*=.57; *I*²=0%) (Figure 10).

Marchand. Effect of the decision to perform hysteroscopy on asymptomatic patients. Am J Obstet Gynecol Glob Rep 2023.

## Discussion

In this meta-analysis, we sought to determine whether hysteroscopy could increase the success rates of ARTs such as in vitro fertilization (IVF), intracytoplasmic sperm injection, and intrauterine insemination. Our analysis demonstrated that performing hysteroscopy before the different assisted conception techniques could increase the clinical pregnancy rate significantly. However, hysteroscopy did not improve the live birth rate, miscarriage rate, fertilization rate, chemical pregnancy rate, multiple pregnancy, number of transferred embryos, or number of oocytes retrieved.

It is notable that a statistically significant difference in the clinical pregnancy rate was observed when performing hysteroscopy before ART, but this did not result in a statistically significant difference in the live birth rate, a result that the authors cannot completely explain. Multiple conditions were diagnosed and treated in the study arms that included hysteroscopy before ART. These treatments included hysteroscopic polypectomy, hysteroscopic myomectomy, lysis of adhesions, and medical treatment for endometritis diagnosed at time of hysteroscopy. One possible explanation for this phenomenon would be the persistence of pathology that would have otherwise prevented implantation later resulting in miscarriage as a result of the inability of our current treatments to truly resolve the condition. Statistically speaking, if this was true for even one of the pathologies, it would explain the lack of significance in the clinical pregnancy data. Another possible explanation is that many patients suffer from >1 pathology, and that after treating a cause found on hysteroscopy (polyps, fibroids, adhesions, endometritis), a second pathology (eg, genetic) then results in miscarriage.

Many previous analyses have found similar results. In 2008, El-Toukhy et al^25^ conducted a meta-analysis that was limited to office hysteroscopy but otherwise considered similar outcomes, and found improvements in almost all outcomes with routine hysteroscopy. They attributed this beneficial role to the ability of hysteroscopy to visualize and treat intrauterine abnormalities including polyps, fibroids, endometritis, and intrauterine adhesions. In researching the percentage of female patients suffering from hysteroscopically correctable uterine pathology, we found several authors estimating that such pathologies are found at the time of hysteroscopy in approximately 50%.^26^, ^27^, ^28^

Endometritis in particular was of interest in many of these studies because many authors have previously referenced the increased sensitivity of hysteroscopic visualization over ultrasound in the diagnosis of chronic endometritis, with some sources citing a >33% increase in sensitivity.^29^^,^^30^ This would lead to greater opportunity for the use of antimicrobial treatments for chronic endometritis in infertile patients given that chronic endometritis is estimated to be a cause in up to 40% of women suffering from infertility worldwide.^30^

Another previous meta-analysis by Chung et al^31^ from 2006 concluded that hysteroscopy could improve IVF outcomes in patients regardless of the presence of hysteroscopic uterine abnormalities. This study, however, was limited to patients who had repeated failures of ARTs as opposed to the routine practice of hysteroscopy.

In more recent reviews, Pundir et al^32^ in 2014 performed a meta-analysis of 6 studies evaluating the efficacy of routine hysteroscopy before the first IVF cycle in infertile women. They concluded that hysteroscopy could significantly increase the clinical pregnancy rate (RR, 1.44; *P*=.01). Although consistent with our findings, this study was limited by significant heterogeneity in all outcomes. In addition, only 1 of the 6 included studies was randomized.

Before this study, the most recent analysis by Mao et al^33^ in 2019 included 3932 patients and showed that hysteroscopy was associated with a better clinical pregnancy rate (*P*<.001) and implantation rate (*P*=.025) compared with the control group. As in our study, they found no significant difference between the hysteroscopy group and the control group in terms of live birth rate and miscarriage rate. This analysis included only 8 studies, only 3 of which were RCTs.

Although our findings were largely consistent with previous analyses, the exact mechanism of improving the clinical pregnancy rate after hysteroscopy is still unclear. Raju et al^23^ suggested that hysteroscopy may treat small intrauterine lesions to increase the pregnancy rate. Shohayeb et al^34^ reported that it was likely that endometrial “scratching” or performing a biopsy could change the features of the endometrium, facilitating embryo implantation. We do not believe that there is any clear consensus on the mechanism of action at this time.

### Strengths and limitations

This was a large meta-analysis that had sufficient evidence to include only RCTs because of the newly published studies since the last analysis. With regard to limitations, 3 of the 8 outcomes were heterogeneous. Although heterogeneity decreases the certainty of evidence according to GRADE (Grading of Recommendations, Assessment, Development and Evaluations) guidelines, we were able to solve the heterogeneity in all cases. This was accomplished by subgroup analysis and the “leave-one-out method,” as described in the Cochrane handbook.^12^

## Conclusion

We found that hysteroscopy performed before different ART procedures could improve the clinical pregnancy rate. A trend was observed toward an increased live birth rate in the hysteroscopy group, but statistical significance was not reached. We did not observe an increase in the miscarriage rate, fertilization rate, chemical pregnancy rate, multiple pregnancy, number of transferred embryos, or number of oocytes retrieved. Thus, we consider that the beneficial role of hysteroscopy should be assessed in further RCTs with more high-quality evidence before considering it a routine procedure in infertile women. Analysis of the cost-effectiveness of routine hysteroscopy before ART treatment would also provide valuable data.
